# Incidence and prevalence of acromegaly in a large US health plan database

**DOI:** 10.1007/s11102-015-0701-2

**Published:** 2016-01-20

**Authors:** Tanya Burton, Elisabeth Le Nestour, Maureen Neary, William H. Ludlam

**Affiliations:** Optum, 950 Winter Street, Waltham, MA 02451 USA; InVentiv Health Clinical, 41 rue des 3 fontanot, 92000 Nanterre, France; Novartis Pharmaceuticals Corporation, One Health Plaza, East Hanover, NJ 07936 USA

**Keywords:** Acromegaly, Incidence, Prevalence, Database analysis

## Abstract

**Purpose:**

Incidence and prevalence estimates of acromegaly in the United States (US) are limited. Most existing reports are based on European data sources. The objective of this study was to estimate the annual incidence and prevalence of acromegaly in a large US managed care population, overall and stratified by age, sex, and geographic region, using data from 2008 to 2012.

**Methods:**

Using administrative claims data, commercial health plan enrollees were identified with acromegaly if they had two or more medical claims with an acromegaly diagnosis code (ICD-9-CM: 253.0×) or one medical claim with an acromegaly diagnosis code in combination with one other claim for a pituitary tumor or pituitary procedure. The first date for an acromegaly-related claim set the index year. Incidence rates for each year were calculated by dividing the number of new acromegaly cases by the calculated person-time at risk. Annual prevalence estimates were calculated by dividing the number with any evidence of acromegaly by the total number of health plan enrollees enrolled for at least 1 day during each calendar year. Incidence and prevalence estimates were stratified by age (0–17, 18–44, 45–64, 65+ years), sex (male, female), and US geographic region of the health plan (Midwest, Northeast, South, West).

**Results:**

Overall annual incidence rates of acromegaly were relatively constant across 2008–2012 with ~11 cases per million person-years (PMPY). Rates increased with age, ranging from 3–8 cases PMPY among children aged 0–17 years old to 9–18 cases PMPY among adults aged 65 and older. Females had 12 cases PMPY on average compared to 10 cases PMPY among men. On average, the Midwest had the lowest incidence rates (7 cases PMPY) compared to the Northeast, South and West (14, 12, and 10 cases PMPY, respectively). The overall annual prevalence of acromegaly was relatively constant across the 5 years from 2008 to 2012 with approximately 78 cases per million each year. Annual prevalence estimates increased with age, ranging from 29–37 cases per million among children aged 0–17 years old to 148–182 cases per million among adults aged 65 years and older. Males and females were similarly affected; each with approximately 77 cases per million each year. The Northeast and South had the highest prevalence estimates (92 and 89 cases per million, respectively); while the estimates for the West and Midwest were lower (65 and 57 cases per million, respectively) each year.

**Conclusion:**

This study examined 5 years of recent data to estimate the incidence and prevalence of acromegaly in a large geographically-diverse managed care population. The incidence rates were higher on average than published rates outside the US (11 vs. 3.3 PMPY), but prevalence estimates were consistent with previous reports. Incidence and prevalence both increased by age, did not differ for males and females, and varied slightly by US geographic region. The age and sex distribution of the selected population matched the known epidemiology of the disease. Using a claims-based approach, this analysis only captured acromegaly cases with an acromegaly-related medical claim. As a result, these estimates may underestimate the incidence and prevalence of acromegaly in US commercial health plans as they did not include individuals who were undiagnosed, in remission, undertreated, or not monitored during the study period. At the same time, these estimates may be viewed as an upper bound on the incidence of acromegaly in the US as the estimates did not include individuals who were in other health plans or uninsured during the study period. Additional evaluations are needed to identify the full extent of acromegaly in the US.

**Electronic supplementary material:**

The online version of this article (doi:10.1007/s11102-015-0701-2) contains supplementary material, which is available to authorized users.

## Introduction

Most incidence and prevalence estimates of acromegaly come from studies conducted in countries outside of the United States (US), often using data from disease registries or national health care systems. Population-based lifetime data are ideal for tracking the development and existence of rare diseases such as acromegaly. However, estimating the presence of acromegaly in any data source is complicated by its insidious nature that changes slowly over time and often mimics many common aging conditions such as diabetes and heart disease. The signs and symptoms of acromegaly are so commonplace among the general population that diagnosis is often delayed an average of 4–7 years after the onset of excessive growth hormone (GH) secretion in adults; but in children, the disease is much less likely to go unnoticed due to their abnormally dynamic growth in physical stature [1].

Most published estimates of acromegaly are fairly consistent with each other but dated. While incidence rates published across Europe, Asia, and New Zealand are remarkably similar, ranging around 2–4 per million per year, the majority are also more than 10 years old [2–11]. Prevalence estimates of acromegaly vary more, ranging from 30 to 100 per million and cover a similar time span with most data collected before 2004 [2–9]. A recent study by Hoskuldsdottir [12] examined Icelandic data from 1955 to 2013 (the most expansive and recent data collected to date) and found slightly higher estimates than other non-US reports (7.7 million new cases of acromegaly per year and 134 per million prevalent cases) [12].

To date, all epidemiologic studies are consistent with respect to the 1:1 sex distribution, mean age of diagnosis around mid-forties, 1:3 micro to macro adenomas, and the approximate rates of surgical success with up to 90 % for micro adenomas and <60 % for macro adenomas when in expert pituitary surgical care [13]. However, little is published about acromegaly in the US. Although there is no universal health care system in the US, large samples of acromegaly patients may be found in private health insurance databases. The objective of this research was to estimate the annual incidence and prevalence of acromegaly from 2008 to 2012 in a large US managed care population using administrative claims data.

## Methods

### Source population

The source population was derived from a large health insurance database, which contains medical and pharmacy claims, and enrollment information from a geographically-diverse group of health plans in the US. Dating back to 1993, the database includes data on more than 123 million US health plan enrollees over time.

The medical claims in the database for professional and facility services include information on diagnoses, reported with *International Classification of Diseases, 9th Revision, Clinical Modification* (ICD-9-CM) diagnosis codes, and procedures, reported with ICD-9-CM, *Current Procedural Terminology, Version 4* (CPT-4), and *Healthcare Common Procedure Coding System* (HCPCS) procedure codes. The medical claims also include site of service codes and health plan- and patient-paid amounts for services received. Outpatient pharmacy claims include national drug codes (NDC), drug dosage form, fill date, health plan- and patient-paid amounts for dispensed medications. All administrative claims data for this study were de-identified and compliant with the provisions of the health insurance portability and accountability act of 1996.

### Acromegaly case identification

The study population included children and adult commercial health plan enrollees in the database between July 1, 2000 and June 30, 2012 (identification period) who met one of the following three acromegaly selection criteria: (1) had at least two medical claims on separate dates with an acromegaly diagnosis code (ICD-9-CM: 253.0×); or (2) had one medical claim with an acromegaly diagnosis code in combination with one medical claim with a pituitary tumor diagnosis code (ICD-9-CM: 237.0×); or (3) had one medical claim with an acromegaly diagnosis code in combination with one medical claim for a pituitary surgery (hypophysectomy) or stereotactic radiosurgery (radiation) procedure (Supplement Table A).

### Person-time at risk

Observation time for acromegaly began on the date of health plan entry. Person-time at risk continued to accrue until the earliest of: acromegaly onset, death, disenrollment from the health plan, or study cut-off, December 31, 2012. The date of acromegaly onset was defined as the first date for an acromegaly-related claim (i.e., a claim with a diagnosis or procedure code for acromegaly, pituitary tumor, hypophysectomy, or radiation) on or after January 1, 2000. The date of acromegaly onset also set the index year. Years prior to the index year were defined as acromegaly-free and years following the index year were defined as having a history of the disease.

### Incidence and prevalence analysis

Annual incidence and prevalence estimates of acromegaly from 2008 to 2012 were derived from the managed care population. Incidence rates were calculated by dividing the number of new acromegaly cases (i.e., no evidence of acromegaly during the 6 months prior to the index claim) by the total time at risk during each calendar year. All incidence rates were reported as the number of cases per one million (1,000,000) person-years at risk. Prevalence was calculated by dividing the number with an acromegaly-related claim during each calendar year or any time prior by the total number continuously enrolled in the health plan for the entire calendar year. Incidence and prevalence estimates were stratified by age (0–17, 18–44, 45–64, 65+ years), sex (male, female), and US geographic region of the health plan (Midwest, Northeast, South, West).

All study variables were summarized descriptively using SAS v9.2 (SAS Institute, Cary, NC).

## Results

**Fig. 1:**
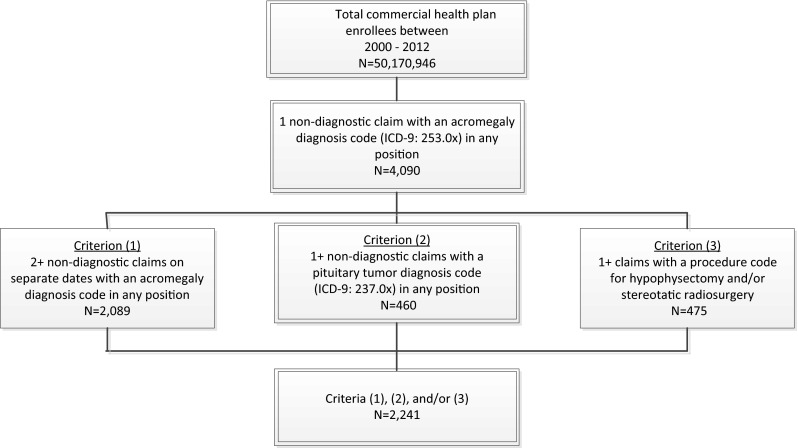
Acromegaly selection

Of the more than 50 million commercial health plan enrollees in the database from 2000 to 2012, 4090 had at least one claim with an acromegaly diagnosis code and 2241 had the additional criteria suggestive of true acromegaly (i.e., additional acromegaly services, a pituitary tumor or procedure) (Fig. 1). Hence, overall prevalence of acromegaly in the database from 2000 to 2012 was estimated to be 45 per million. These subjects had a mean age of 41 years and a near equal sex distribution (48 % males vs. 52 % females). The geographic distribution mirrored the health plan with the majority in the South (55 %) and Midwest (22 %), and smaller proportions in the West (13 %) and Northeast (10 %).

### Incidence and prevalence results

**Fig. 2:**
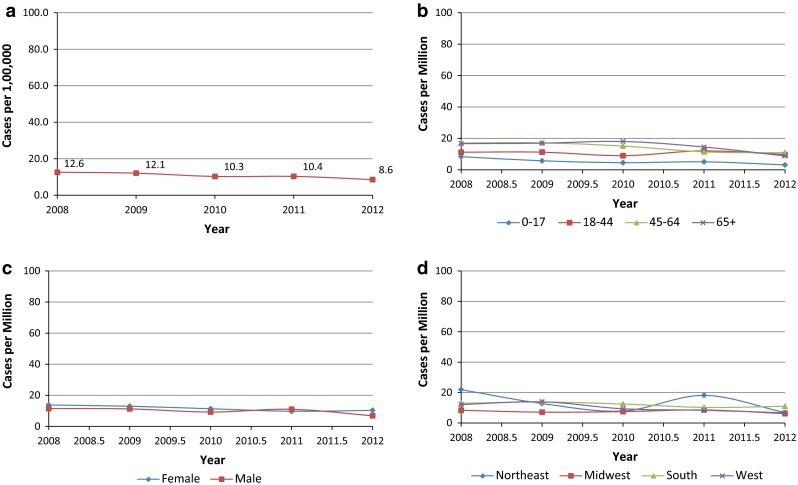
**a** Incidence—overall. **b** Incidence by age. **c** Incidence by sex. **d** Incidence by geographic region

Annual incidence rates of acromegaly were relatively constant across 2008–2012 with an overall rate of approximately 11 cases per million person-years (PMPY) (Fig. 2a and Supplement Table B). Rates increased with age, ranging from 3–8 cases PMPY among children aged 0–17 years old to 9–18 cases PMPY among adults 65 years and older (Fig. 2b). Males and females were similarly affected over time (Fig. 2c). Females had 12 cases PMPY on average compared to 10 cases PMPY among men. On average, Midwest health plans had the lowest incidence rates (7 cases per million PY) compared to health plans in the Northeast (14 cases PMPY), South (12 cases PMPY), and West (10 cases PMPY) (Fig. 2d).

**Fig. 3:**
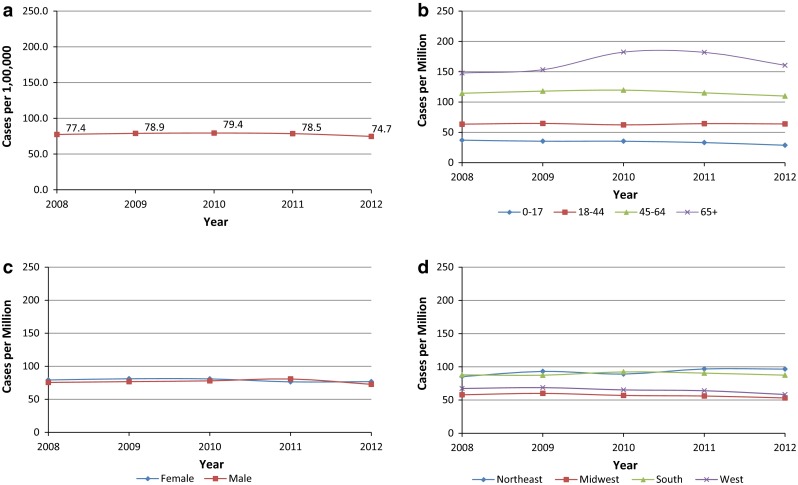
**a** Prevalence—overall. **b** Prevalence by age. **c** Prevalence by sex. **d** Prevalence by geographic region

Prevalence estimates for acromegaly were also fairly constant across the 5 years, with approximately 78 cases per million each year (Fig. 3a and Supplement Table C). Annual prevalence estimates increased with age, ranging from 29–37 cases per million among children aged 0–17 years to 148–182 cases per million among adults aged 65 years and older (Fig. 3b). As with incidence, prevalence estimates for males and females were similar, each with approximately 77 cases per million each year (Fig. 3c). Health plans in the Northeast and South had the highest prevalence (92 and 89 cases per million, respectively), while health plans in the West and Midwest were lower (65 and 57 cases per million, respectively) each year (Fig. 3d).

## Discussion

To our knowledge, this is the first study to assess the incidence and prevalence of acromegaly in a large geographically-diverse managed care population in the US. This study used administrative claims data to estimate the incidence and prevalence of acromegaly between 2008 and 2012. The incidence rates were higher on average than published rates outside the US (11 vs. 3.3 per million person-years), but prevalence estimates were consistent with previous reports. Overall, the incidence and prevalence estimates increased by age, did not differ for males and females, and varied by US geographic region. The age and sex distribution of the selected population matched the known epidemiology of the disease.

It is not surprising that our incidence rates differed from studies outside the US, given the vast differences in the methods used to collect health data around the world. There are several reasons that may explain the difference observed between our incidence estimates and the non-US studies. First, the sample of commercially-insured health plan enrollees in the database primarily includes working-age adults in the US who are more likely to be near the mean age of disease onset than the national populations included in non-US studies. Second, since administrative claims data are collected for the purposes of billing rather than research, it is possible that a diagnosis code on a medical claim may not indicate the presence of actual disease. Although only non-diagnostic medical claims were used to identify acromegaly (claims from laboratories and diagnostic testing centers may include “rule-out” procedures for diagnoses not yet confirmed), it is still possible that acromegaly may have been miscoded or misidentified on claims, which could have increased the reported incidence rates. Third, this analysis required individuals to have at least one health care service to be included in the incidence calculations, which suggests that the reported incidence rates may have been lower if the population had included healthier individuals with no health care use as well. At the same time, this analysis required at least two medical claims to identify acromegaly cases, which suggests that the reported incidence may have been even higher as the estimates did not identify individuals with acromegaly who were undiagnosed, in remission, undertreated, or not monitored during the study period. Other limitations to consider when interpreting these results include the source population and its generalizability. As noted above, the study data came from a geographically-diverse managed care population, which means the results are primarily applicable to populations that receive their care through similar delivery systems across the US. However, this still leaves out a significant proportion of the US population, many of whom have other forms of health insurance such as Medicare, Medicaid, Tricare, or none at all. In comparison, non-US surveys typically are based on disease registries or health systems that collect extensive national data like Finland and New Zealand or offer individuals ongoing access to medical coverage throughout life such as the National Health Service (NHS) in the United Kingdom [2, 11]. In spite of these differences, this study found US prevalence estimates that fit within the range of prior research. Although given the study’s selection criteria, they more likely estimate the prevalence of acromegaly patients with active disease.

To calculate the incidence and prevalence of acromegaly correctly it is important to know not only who was diagnosed, but also when they were diagnosed and the current disease status (e.g., active vs. inactive disease). While US health plan databases have the ability to track individuals longitudinally, their populations can change frequently as individuals enroll and disenroll from health plans over time. As a result, acromegaly-related care received outside of the health plan is not always included in the database or may be excluded when analyzing individual cuts of the data (e.g., when using a subset of data between 2008 and 2012). This study sought to overcome these limitations by requiring at least a 6-month continuous enrollment period and including all medical claims in the database dating back to January 2000 to check for acromegaly history regardless of enrollment status. Lastly, to identify our sample without clinical data such as the date of diagnosis, substantial emphasis was placed on multiple pathways to acromegaly case identification. However, no medical chart review was conducted to validate our claims-based definition. Given that acromegaly is challenging to diagnose and cannot be confirmed using claims data alone, future research should include clinical data from medical charts or electronic health records to validate the algorithm used to identify acromegaly patients in administrative claims databases.

## Conclusion

This study examined 5 years of recent data to estimate the incidence and prevalence of acromegaly in a large geographically-diverse managed care population in the US. The incidence rates were higher on average than published rates outside the US (11 vs. 3.3 per million person-years), but prevalence estimates (~78 cases per million each year) were consistent with previous reports. Overall, the incidence and prevalence estimates increased by age, did not differ for males and females, and varied slightly by US geographic region. The age and sex distribution of the selected population matched the known epidemiology of the disease. Using a claims-based approach, this analysis only captured acromegaly cases with an acromegaly-related medical claim. As a result, these estimates may underestimate the incidence and prevalence of acromegaly in US commercial health plans as they did not include individuals who were undiagnosed, in remission, undertreated, or not monitored during the study period. At the same time, these estimates may be viewed as an upper bound on the incidence of acromegaly in the US as the estimates did not include individuals who were in other health plans or uninsured during the study period. While the claims-based algorithm was not validated with a medical chart review, this study did find data that matched the known epidemiology of the disease. Additional evaluations are needed to identify the full extent of acromegaly in the US.

## Electronic supplementary material

Below is the link to the electronic supplementary material.

Supplementary material 1 (DOCX 15 kb)

Supplementary material 2 (DOCX 21 kb)

Supplementary material 3 (DOCX 20 kb)
