# Supplementary material for: Incidence and prevalence of acromegaly in a large US health plan database
Source: Pituitary. 2016 Jan 20;19:262–7. doi: 10.1007/s11102-015-0701-2 (PMC4858553; doi:10.1007/s11102-015-0701-2)
Supplement: Supplementary file 1 — Supplementary material 1 (DOCX 15 kb) [file 11102_2015_701_MOESM1_ESM.docx]

Supplement Table A. Codes for Acromegaly-related Procedures

| **Code Type** | **Code** | **Description** | **Condition/Procedure** |
| --- | --- | --- | --- |
| CPT | 61546 | Craniotomy for hypophysectomy or excision of pituitary tumor, intracranial approach | Hypophysectomy |
|  | 61548 | Hypophysectomy or excision of pituitary tumor, transnasal or transseptal approach, nonstereotactic |  |
|  | 62165 | Neuroendoscopy, intracranial; with excision of pituitary tumor, transnasal or trans-sphenoidal approach |  |
| ICD-9 Procedure | 07.61 | Partial excision of pituitary gland, transfrontal approach |  |
|  | 07.62 | Partial excision of pituitary gland, transsphenoidal approach |  |
|  | 07.63 | Partial excision of pituitary gland, unspecified approach |  |
|  | 07.64 | Total excision of pituitary gland, transfrontal approach |  |
|  | 07.65 | Total excision of pituitary gland, transsphenoidal approach |  |
|  | 07.68 | Total excision of pituitary gland, other specified approach |  |
|  | 07.69 | Total excision of pituitary gland, unspecified approach |  |
| CPT | 61796 | Stereotactic radiosurgery (particle beam, gamma ray, or linear accelerator); 1 simple cranial lesion | Cranial stereotactic radiosurgery |
|  | 61797 | Stereotactic radiosurgery (particle beam, gamma ray, or linear accelerator); each additional cranial lesion, simple (List separately in addition to code for primary procedure) |  |
|  | 61798 | Stereotactic radiosurgery (particle beam, gamma ray, or linear accelerator); 1 complex cranial lesion |  |
|  | 61799 | Stereotactic radiosurgery (particle beam, gamma ray, or linear accelerator); each additional cranial lesion, complex (List separately in addition to code for primary procedure) |  |
|  | 77371 | Radiation treatment delivery, stereotactic radiosurgery (SRS), complete course of treatment of cranial lesion(s) consisting of 1 session; multi-source Cobalt 60 based |  |
|  | 77372 | Radiation treatment delivery, stereotactic radiosurgery (SRS), complete course of treatment of cranial lesion(s) consisting of 1 session; linear accelerator based |  |
