# Supplementary material for: Incidence and prevalence of acromegaly in a large US health plan database
Source: Pituitary. 2016 Jan 20;19:262–7. doi: 10.1007/s11102-015-0701-2 (PMC4858553; doi:10.1007/s11102-015-0701-2)
Supplement: Supplementary file 2 — Supplementary material 2 (DOCX 21 kb) [file 11102_2015_701_MOESM2_ESM.docx]

Supplement Table B. 2008 – 2012 Annual Incidence of Acromegaly in a Large US Health Plan Database

|  | **2008** | | | **2009** | | | **2010** | | | **2011** | | | **2012** | | |
| --- | --- | --- | --- | --- | --- | --- | --- | --- | --- | --- | --- | --- | --- | --- | --- |
|  | **Events** | **Person-years** | **Rate per 100,000** | **Events** | **Person-years** | **Rate per 100,000** | **Events** | **Person-years** | **Rate per 100,000** | **Events** | **Person-years** | **Rate per 100,000** | **Events** | **Person-years** | **Rate per 100,000** |
| Acromegaly | 116 | 9,199,158 | 1.261 | 109 | 9,006,395 | 1.210 | 88 | 8,563,386 | 1.028 | 89 | 8,601,055 | 1.035 | 73 | 8,531,645 | 0.856 |
| Age |  |  |  |  |  |  |  |  |  |  |  |  |  |  |  |
| 0-17 | 18 | 2,154,441 | 0.835 | 12 | 2,097,760 | 0.572 | 9 | 2,000,323 | 0.450 | 10 | 1,965,833 | 0.509 | 6 | 1,917,788 | 0.313 |
| 18-44 | 42 | 3,751,284 | 1.120 | 41 | 3,645,109 | 1.125 | 31 | 3,455,800 | 0.897 | 42 | 3,480,576 | 1.207 | 34 | 3,509,619 | 0.969 |
| 45-64 | 49 | 2,873,167 | 1.705 | 49 | 2,850,682 | 1.719 | 42 | 2,775,097 | 1.513 | 32 | 2,810,047 | 1.139 | 30 | 2,764,492 | 1.085 |
| 65+ | 7 | 420,266 | 1.666 | 7 | 412,845 | 1.696 | 6 | 332,168 | 1.806 | 5 | 344,599 | 1.451 | 3 | 339,747 | 0.883 |
| Sex |  |  |  |  |  |  |  |  |  |  |  |  |  |  |  |
| Female | 64 | 4,655,645 | 1.375 | 59 | 4,557,459 | 1.295 | 49 | 4,325,577 | 1.133 | 42 | 4,331,309 | 0.970 | 44 | 4,270,811 | 1.030 |
| Male | 52 | 4,543,513 | 1.144 | 50 | 4,448,936 | 1.124 | 39 | 4,237,809 | 0.920 | 47 | 4,269,746 | 1.101 | 29 | 4,260,834 | 0.681 |
| Geographic region |  |  |  |  |  |  |  |  |  |  |  |  |  |  |  |
| Northeast | 21 | 957,718 | 2.193 | 12 | 938,418 | 1.279 | 7 | 890,227 | 0.786 | 16 | 879,810 | 1.819 | 6 | 881,315 | 0.681 |
| Midwest | 20 | 2,395,119 | 0.835 | 16 | 2,252,700 | 0.710 | 16 | 2,145,643 | 0.746 | 19 | 2,213,431 | 0.858 | 14 | 2,282,167 | 0.613 |
| South | 58 | 4,488,309 | 1.292 | 61 | 4,422,090 | 1.379 | 52 | 4,172,639 | 1.246 | 42 | 4,115,131 | 1.021 | 43 | 3,913,021 | 1.099 |
| West | 17 | 1,403,667 | 1.211 | 20 | 1,435,085 | 1.394 | 13 | 1,395,831 | 0.931 | 12 | 1,436,394 | 0.835 | 10 | 1,500,214 | 0.667 |
