# Supplementary material for: Incidence and prevalence of acromegaly in a large US health plan database
Source: Pituitary. 2016 Jan 20;19:262–7. doi: 10.1007/s11102-015-0701-2 (PMC4858553; doi:10.1007/s11102-015-0701-2)
Supplement: Supplementary file 3 — Supplementary material 3 (DOCX 20 kb) [file 11102_2015_701_MOESM3_ESM.docx]

Supplement Table C. 2008 – 2012 Annual Prevalence of Acromegaly in a Large US Health Plan Database

|  | **2008** | | | **2009** | | | **2010** | | | **2011** | | | **2012** | | |
| --- | --- | --- | --- | --- | --- | --- | --- | --- | --- | --- | --- | --- | --- | --- | --- |
|  | **Events** | **Person-years** | **Rate per 100,000** | **Events** | **Person-years** | **Rate per 100,000** | **Events** | **Person-years** | **Rate per 100,000** | **Events** | **Person-years** | **Rate per 100,000** | **Events** | **Person-years** | **Rate per 100,000** |
| Acromegaly | 654 | 8,452,018 | 7.738 | 663 | 8,401,369 | 7.892 | 628 | 7,910,203 | 7.939 | 630 | 8,022,220 | 7.853 | 596 | 7,979,649 | 7.469 |
| Age |  |  |  |  |  |  |  |  |  |  |  |  |  |  |  |
| 0-17 | 74 | 1,996,083 | 3.707 | 70 | 1,982,868 | 3.530 | 66 | 1,869,373 | 3.531 | 61 | 1,843,527 | 3.309 | 52 | 1,811,453 | 2.871 |
| 18-44 | 214 | 3,371,698 | 6.347 | 216 | 3,337,462 | 6.472 | 195 | 3,130,350 | 6.229 | 207 | 3,215,859 | 6.437 | 207 | 3,241,685 | 6.386 |
| 45-64 | 308 | 2,691,868 | 11.442 | 318 | 2,696,063 | 11.795 | 312 | 2,608,869 | 11.959 | 305 | 2,649,603 | 11.511 | 288 | 2,621,313 | 10.987 |
| 65+ | 58 | 392,369 | 14.782 | 59 | 384,976 | 15.326 | 55 | 301,611 | 18.235 | 57 | 313,231 | 18.197 | 49 | 305,198 | 16.055 |
| Sex |  |  |  |  |  |  |  |  |  |  |  |  |  |  |  |
| Female | 340 | 4,291,721 | 7.922 | 346 | 4,268,641 | 8.106 | 324 | 4,007,283 | 8.085 | 309 | 4,046,903 | 7.636 | 307 | 4,001,340 | 7.672 |
| Male | 314 | 4,160,297 | 7.548 | 317 | 4,132,728 | 7.671 | 304 | 3,902,920 | 7.789 | 321 | 3,975,317 | 8.075 | 289 | 3,978,309 | 7.264 |
| Geographic region |  |  |  |  |  |  |  |  |  |  |  |  |  |  |  |
| Northeast | 76 | 891,180 | 8.528 | 82 | 880,828 | 9.309 | 74 | 830,341 | 8.912 | 80 | 826,270 | 9.682 | 80 | 828,510 | 9.656 |
| Midwest | 127 | 2,193,272 | 5.790 | 125 | 2,082,082 | 6.004 | 114 | 2,003,666 | 5.690 | 117 | 2,086,913 | 5.606 | 115 | 2,163,701 | 5.315 |
| South | 363 | 4,129,810 | 8.790 | 365 | 4,177,050 | 8.738 | 356 | 3,853,594 | 9.238 | 347 | 3,832,896 | 9.053 | 318 | 3,636,291 | 8.745 |
| West | 88 | 1,306,239 | 6.737 | 91 | 1,324,487 | 6.871 | 84 | 1,287,597 | 6.524 | 86 | 1,343,324 | 6.402 | 83 | 1,422,299 | 5.836 |
